# Glucose and fatty acids catabolism during in vitro decidualization of human endometrial stromal cells

**DOI:** 10.1007/s10815-022-02637-3

**Published:** 2022-10-29

**Authors:** Ana Cecilia Mestre Citrinovitz, Jana Hauke, Julia Jauckus, Claus-Dieter Langhans, Kathrin Schwarz, Markus Zorn, Thomas Strowitzki, Juergen G. Okun, Ariane Germeyer

**Affiliations:** 1 Department of Gynecological Endocrinology and Fertility Disorders, Women’s Hospital, Ruprecht-Karls University of Heidelberg, INF 440, 69120 Heidelberg, Germany; 2 Centro de Investigaciones en Toxicología Ambiental y Agrobiotecnología del Comahue (CITAAC), Consejo Nacional de Investigaciones Científicas y Tecnológicas (CONICET), Universidad Nacional del Comahue, Neuquén, Argentina; 3 Metabolic Laboratory and Newborn Screening, Dietmar-Hopp-Metabolic Center, University Children’s Hospital, Heidelberg University Hospital, INF 669, 69120 Heidelberg, Germany; 4 Central Laboratory, University Hospital, Ruprecht-Karls University of Heidelberg, INF 671, 69120 Heidelberg, Germany

**Keywords:** Human endometrial stromal cells, Decidualization, Glucose, Fatty acids, β-Oxidation pathway, TCA cycle

## Abstract

The differentiation of endometrial stromal cells, named decidualization, is essential for the proper formation of the materno-fetal interphase. One important feature of decidualization is the increased glucose consumption and its utilization by endometrial cells to produce energy. Besides glucose, fatty acids are another important energy source for living cells and it has been described that endometrial stromal cells rely on the proper function of the oxidation of fatty acids for the correct decidualization. It is, however, unknown whether the turn-over of fatty acid degradation is modified during decidualization. Furthermore, it is also unknown how the final products of glucose and fatty acid catabolism are related to the function of the tricarboxylic acid cycle for the efficient ATP production. In this study, we evaluated the content levels of different intermediate metabolites and the expression of the key enzymes related to the degradation of glucose and fatty acids during the in vitro decidualization of human endometrial stromal cells. Our results suggest that human endometrial stromal cells undergo energetic metabolic changes during decidualization and that decidualizing and non-decidualizing cells differ in the level of activation of different metabolic pathways and, probably, in the use of intermediate metabolites.

**Supplementary Information:**

The online version contains supplementary material available at 10.1007/s10815-022-02637-3.

## Introduction

During the secretory phase of the menstrual cycle, fibroblastic-like endometrial stromal cells (ESC) differentiate into enlarged decidual cells in response to the circulating levels of progesterone and the intracellular increased levels of cyclic adenosine monophosphate (cAMP) [1]. The differentiation of ESC, known as decidualization, is essential for the proper interaction of the endometrium with the implanting blastocyst and is of great importance to ensure the formation of the materno-fetal interface [2, 3]. Decidualization compromises global changes in gene expression and a complex change of the morphology and function of ESC [4, 5]. For example, in contrast to ESC, decidual cells have their endoplasmic reticulum greatly developed and are highly secretory, producing prolactin (PRL), one of the main secretory products of decidual cells, which is commonly used as a decidualization marker [2, 6–8].

Due to the molecular and cellular changes that decidualization involves, ESC differentiation is a process with a high energetic and metabolic demand. Therefore, another important feature of decidualization is the increased consumption of glucose, as the main energy source [9]. In general, glucose is incorporated into cells through glucose transporters (GLUT 1-4) (See Supplementary Fig. S1). It has been described that GLUT1 and GLUT3 are expressed in the human endometrium and GLUT1 expression is increased during the secretory phase of the menstrual cycle and also during in vitro decidualization of human and murine ESC [9, 10]. Furthermore, the inhibition of glucose transporters by cytochalasin B lead to a reduced glucose uptake and decreased expression of PRL during the in vitro decidualization of human ESC [9]. In addition, it has been described that progesterone increases GLUT1 expression and glucose uptake in murine ESC [10].

Once glucose is incorporated into the cells, it is subsequently degraded by glycolysis or the pentose phosphate pathway (PPP), which are the main pathways of glucose catabolism in eukaryotic cells. Glycolysis involves glucose degradation with ATP, pyruvic acid, and NADH reducing agent as the direct products, while the PPP pathway involves glucose degradation for the production of NADPH reducing agent and of ribose-5-phosphate, the precursors for ribonucleosides (RN) and deoxyribonucleosides (DRN) synthesis. Regarding glucose utilization by ESC, it has been described that aerobic glycolysis-related genes are induced during artificial decidualization in mice, indicating the existence of a Warburg-like effect during decidua formation [11]. Furthermore, these authors also showed that the knockdown of pyruvate kinase M2 (Pkm2), the enzyme that catalyzed pyruvic acid formation during glycolysis, attenuates the induction of a murine decidual marker gene during the in vitro decidualization of primary mouse ESC [11]. In relation to the PPP, it has been described that in mice the pharmacological inhibition of the PPP pathway interferes with the correct in vitro decidualization of ESC and reduces the extend of decidua obtained by artificial decidualization [12]. This result has been reinforced by in vitro experiments in which the knock-down of the PPP-rate-limiting enzyme, glucose-6-phosphate dehydrogenase (G6PD), by specific short hairpin RNA transfection of ESC lead to reduced PRL expression during in vitro decidualization [12]. As shown for *GLUT1*, it has been demonstrated that the expression of *G6PD* in the endometrium is regulated by progesterone [9, 13].

Besides glucose, another important energy source used by cells is fatty acids, which are metabolized in the mitochondria through the β-oxidation pathway. Free long-chain fatty acids are shuttled inside the mitochondria by a translocase with the essential contribution of the enzymes carnitine palmitoyl transferase 1 (CPT1) and 2 (CPT2) [14–16] (See Supplementary Fig. S1). The final products of the β-oxidation of fatty acids are acetyl coenzyme A (acetyl-CoA) and reducing agents (NADH and FADH_2_). Furthermore, several other intermediate lipid intermediates with varying chain lengths, are also generated during this process. In the endometrium, it has been described that the pharmacological inhibition and the short hairpin RNA-mediated silencing of CPT1 affect decidualization of human ESC, highlighting the importance of the β-oxidation pathway for endometrial function [17].

In terms of further glucose and fatty acid catabolism, the pyruvic acid resulted from glycolysis and the acetyl-CoA resulted from the β-oxidation pathway, converge in the tricarboxylic acid cycle (TCA cycle, also known as Krebs cycle), which also takes place inside the mitochondria. The TCA cycle involves several bi-directional enzymes that generate different intermediate metabolites and reducing agents (NADH and FADH_2_) [18]. The intermediate metabolites interconnect the TCA cycle with other metabolic pathways of the cell and, in the presence of molecular oxygen, the reducing agents are used in the mitochondrial electron transport chain to produce high amounts of ATP. Until now, the content level of the intermediate metabolites of the TCA cycle and the expression levels of the key enzymes of the pathway has not been evaluated during the decidualization of human ESC (HESC). Therefore, the aim of this study was to evaluate the changes in the catabolic pathways of glucose and fatty acids, including the intermediate metabolites and the key enzymes of the TCA cycle, to get more insight into the energetic metabolism during in vitro decidualization of primary HESC.

## Materials and methods

### Cell isolation, culture, and in vitro decidualization

#### Cell isolation and culture

Endometrial biopsies from the mid-late proliferative phase were obtained from healthy regularly-cycling women (33.6 ± 2.2 years old) without hormonal therapies, no endometrial abnormalities, and no endometriosis at diagnostic laparoscopy. To isolate primary human ESC (HESC), endometrial biopsies were cut into small pieces and digested with collagenase (Gibco, Karlsruhe, Germany) and hyaluronidase (Sigma–Aldrich, Taufkirchen, Germany) at 37 °C for 60 to 90 min as previously described [7]. To remove epithelial cells, cell suspensions were filtered through a 40 μm mesh. Isolated HESC were cultured in a 3:1 mixture of DMEM (21063, Gibco, Karlsruhe, Germany) and MCDB-105 (M6395, Sigma–Aldrich, Taufkirchen, Germany) without phenol red, supplemented with 10% v/v charcoal/dextran treated fetal bovine serum (FBS, HyClone, GE Healthcare Europe, Freiburg, Germany). Cell passages number 2 to 4 were used for the experiments described below.

#### In vitro decidualization

HESC were trypsinized, counted, and plated in 6-well plates (150,000 cells/well). After 24–48 h, when cells had reached 80% confluency, cells were rinsed with PBS and 2 ml of fresh 2% v/v FBS medium supplemented with a decidualization cocktail containing 1 μM medroxyprogesterone acetate (MPA), 10 nM estradiol (E) and 0.5 mM 8-Bromo-cAMP (8-Br-cAMP) (D, decidualizing) or vehicle solutions (ND, non-decidualizing) were added to each well as previously performed [8]. Cell culture medium supplemented with the decidualization cocktail or vehicle solutions was changed every 2 days. At day 6 of the decidualization treatment, cell supernatants (SN) were collected for the quantification of PRL levels and cell samples were collected in TRIzol reagent for total RNA extraction or in RIPA buffer for metabolite analysis.

### Prolactin quantification

Prolactin from cell culture supernatant was quantified at the central laboratory of Heidelberg University Clinic using a double sandwich CLIA assay on a CENTAUR XPT platform (Siemens Healthineers, Eschborn, Germany) according to the manufacturer’s instruction.

### Metabolite analysis

Cells were collected in RIPA buffer supplemented with EDTA-free Complete Protease Inhibitor (Roche, Mannheim, Germany) and PhosSTOP Phosphatase Inhibitor Cocktail (Roche, Mannheim, Germany) after 6 days of the decidualization treatment. The obtained cell homogenates were quantified with the BCA kit (23227, Thermo Scientific, Life Technologies GmbH, Darmstadt, Germany) according to the manufacturer’s instructions.

#### Electrospray ionization-tandem mass spectrometry (ESI-MS/MS)

Acylcarnitines were determined in cell homogenates by ESI-MS/MS according to a modified method previously described [19]. Briefly, 5 μl of cell homogenates were dried overnight at room temperature on a 4.7 mm filter paper punch. The next day, dried cell homogenates were extracted from the filter paper with 100 μl of deuterium-labeled standard solution in methanol. After 20 min, extracted cell homogenates were centrifuged at 3220 × g_max_ for 2 min and the extract was collected in a 96-well microtiter plate (Greiner, Kremsmünster, Austria) and evaporated until dryness. Samples were reconstituted in 60 μl of 3 N HCl/butanol, placed in sealed microtiter plates, and incubated at 65 °C for 15 min. The resulting mixtures were evaporated until dryness and each residue was finally reconstituted in 150 μl of a solvent containing acetonitrile/water (50:50 v/v) prior to measurement [19]. Measurement was performed in a Waters Xevo TQD tandem mass spectrometer (Micromass/Waters Eschborn, Germany) equipped with an electrospray ion source and a Micromass MassLynx data system.

#### Gas chromatography-mass spectroscopy (GC-MS)

For the determination of TCA cycle metabolites, 40 μl of cell homogenates was used for liquid-liquid extraction. Briefly, 100 μl of d4-nitrophenol (1.25 mM, Cambridge Isotope Laboratories, Inc., Tewksbury, MA, USA) and 200 μl of d3-lactic acid (1 mM, C/D/N Isotopes, Inc., Quebec, Canada) were added as internal standards. For determination of oxoglutaric acid, 100 μl of pentafluorobenzyl hydroxylamine (100 mM, Sigma-Aldrich Chemie GmbH, Taufkirchen, Germany) was added as oximation reagent. Samples were acidified with 300 μl of 5 M HCl and were kept at room temperature for 1 h to complete oximation. Then, solid sodium chloride was added and the resulting solution was extracted twice with 5 ml of ethyl acetate each time. The combined ethyl acetate fractions were dried over sodium sulfate for 30 min and then dried down at 40 °C under a stream of nitrogen. After complete removal of the solvent, samples were derivatized with 50 μl N-methyl-N-(trimethylsilyl) heptafluorobutyramide (MSHFBA, Macherey-Nagel, Düren, Germany) at 60 °C for 1 h. For GC/MS analysis, the quadrupole mass spectrometer MSD 5977A (Agilent, Santa Rosa, CA, USA) was run in the selective ion-monitoring mode with electron impact ionization. Gas chromatographic separation was achieved on a capillary column (DB-5MS, 30 m × 0.25 mm; film thickness: 0.25; J&W Scientific, Folsom, CA, USA) using helium as a carrier gas. A volume of 1 μl of the derivatized sample was injected in splitless mode. GC temperature parameters were 60 °C for 1 min, ramp 50 °C/min to 150 °C, ramp 4 °C/min to 259 °C, and hold at 300 °C for 2 min. Injector temperature was set to 280 °C and interface temperature to 290 °C. Fragment ions for quantification were m/z 245 (fumaric acid), m/z 233 (malic acid), m/z 247 (succinic acid), m/z 340 (pyruvic acid), m/z 470 (oxoglutaric acid), m/z 200 (d_4_-nitrophenol), m/z 219 (lactic acid), and m/z 222 (d_3_-lactic acid). A dwell time of 50 ms was used.

### Gene expression assays

Cells were collected in TRIzol reagent and total RNA extraction and cDNA synthesis was performed as previously described [7, 8]. Real time-PCR (RT-PCR) was performed using tagman primers (listed in Supplemental Table S1) (Life Technologies GmbH, Darmstadt, Germany) and TaqMan universal PCR master mix (Applied Biosystems, Life Technologies GmbH, Darmstadt, Germany). RT-PCR reactions were run in the fast forward 7500 real-time PCR-system (Applied Biosystems, Life Technologies GmbH, Darmstadt, Germany), and results were analyzed according to the ΔΔCt method as previously performed [7, 8]. RPLP0 was used as internal control. Normalized Ct values (ΔCt normalized to RPLP0 Ct value) were used for statistical analysis.

### Statistical analysis

Paired student’s *t*-test was used for statistical analysis. Differences between treatments were considered significant when *p* < 0.05. Statistical analysis was carried out using GraphPad Prim 5.0 (GraphPad Software Inc., La Jolla, CA, USA). Results are expressed as mean ± SEM.

## Results

### In vitro decidualization of HESC

**Fig. 1:**
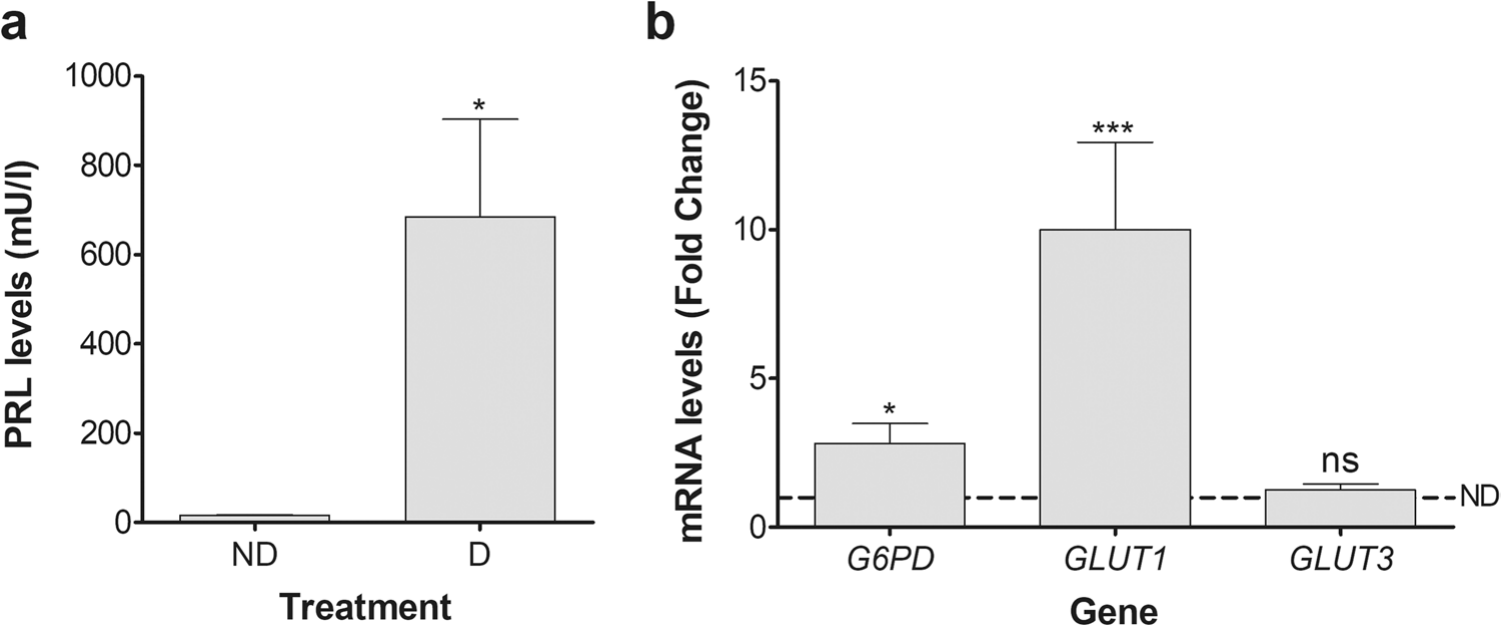
Validation of primary HESC in vitro decidualization. HESC were cultivated for 6 days in 2%-FBS medium containing MPA, E, and 8-Br-cAMP (D cells) or in 2%FBS medium containing vehicle solutions (ND cells). **a** PRL levels in cell culture supernatant. Data represents mean mU/l ± SEM (*n* = 5). **b** *G6PD*, *GLUT1*, and *GLUT3* mRNA expression levels. Data represent mean fold change compared to ND cells (dotted line, fold change = 1.0) ± SEM (*n* = 5). *p < 0.05 and ***p < 0.001 ns, non-significant (compared to ND cells). HESC, human endometrial stromal cells; D, decidualizing; ND, non-decidualizing; MPA, medroxyprogesterone acetate; E, estradiol; 8-Br-cAMP, 8-bromo-cyclic adenosine monophosphate; PRL, Prolactin; GLUT, glucose transporter; and G6PD, glucose-6-phosphate dehydrogenase

As a first step to define the differences in glucose catabolism between decidualizing (D) and non-decidualizing (ND) cells, we validated the decidualization status of primary HESC when treated with the decidualization cocktail. As expected, PRL protein levels in HESC culture supernatant were significantly increased in D compared to ND cells (Fig. 1a). In addition, we evaluated the mRNA expression levels of glucose transporters *GLUT1*, *GLUT3*, and of the PPP enzyme *G6PD*. The expression of *GLUT1* and *G6PD* was highly upregulated in D cells, while the expression of *GLUT3* had no differences between D and ND cells (Fig. 1b).

### Evaluation of the β-oxidation pathway in decidualizing HESC

**Fig. 2:**
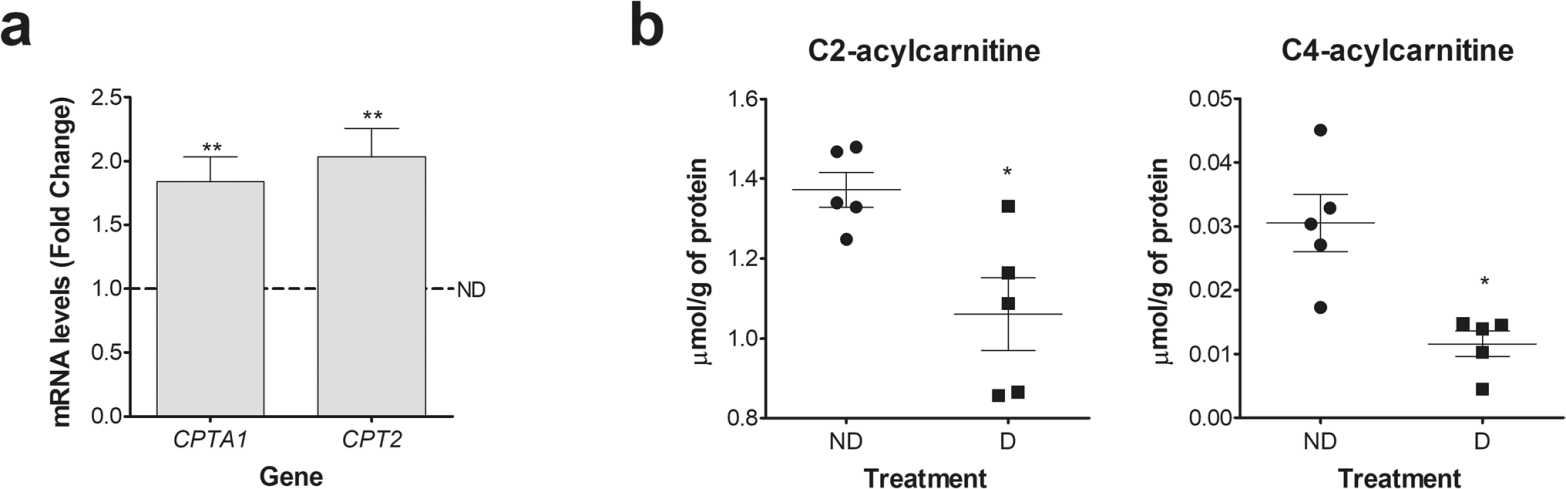
In vitro decidualization of HESC modifies the expression levels of lipid transferases and the content of metabolic intermediates associated with the β-oxidation pathway. HESC were cultivated for 6 days in 2%-FBS medium containing MPA, E, and 8-Br-cAMP (D cells) or in 2%FBS medium containing vehicle solutions (ND cells). **a** *CPTA1* and *CPT2* mRNA expression levels. Data represent mean fold change compared to ND cells (dotted line, fold change = 1.0) ± SEM (*n* = 5). **b** C2- and C4-acylcarnitine levels. Data represent mean μmol/g of total protein (*n* = 5). *p < 0.05 and **p < 0.01 (compared to ND cells). HESC, human endometrial stromal cells; D, decidualizing; ND, non-decidualizing; MPA, medroxyprogesterone acetate; E, estradiol; 8-Br-cAMP, 8-bromo-cyclic adenosine monophosphate; and CPT, carnitine Palmitoyl Transferase

Once confirmed that the HESC were behaving as expected upon the treatment with the decidualization cocktail, we moved forward to evaluate the mRNA expression levels of *CPTA1* and *CPT2*. The expression levels of these transferases were upregulated in D cells compared to ND cells (Fig. 2a). Fatty acids need to be bonded to carnitine to be able to move inside or outside the mitochondria. The presence of fatty acids inside the mitochondria is needed for their oxidation and the presence of fatty acids outside the mitochondria contributes to their availability in the cytoplasm. These carnitine-bonded fatty acids are called acylcarnitines and the length of the chain of the fatty acid of origin will determine the length of the carbon (C) chain (short, medium, or long-chain) of the acylcarnitine. We evaluated the content levels of different acylcarnitines as an equivalent of the intermediate and final products of the β-oxidation pathway and we found that the concentrations of acetyl (C2)- and butyryl (C4)-acylcarnitines were decreased in D compared to ND cells (Fig. 2b). We found no differences between D and ND cells in the content level of other medium-chain acylcarnitines evaluated (data not shown).

### Pyruvic and lactic acid and evaluation of the TCA cycle in decidualizing HESC

**Fig. 3:**
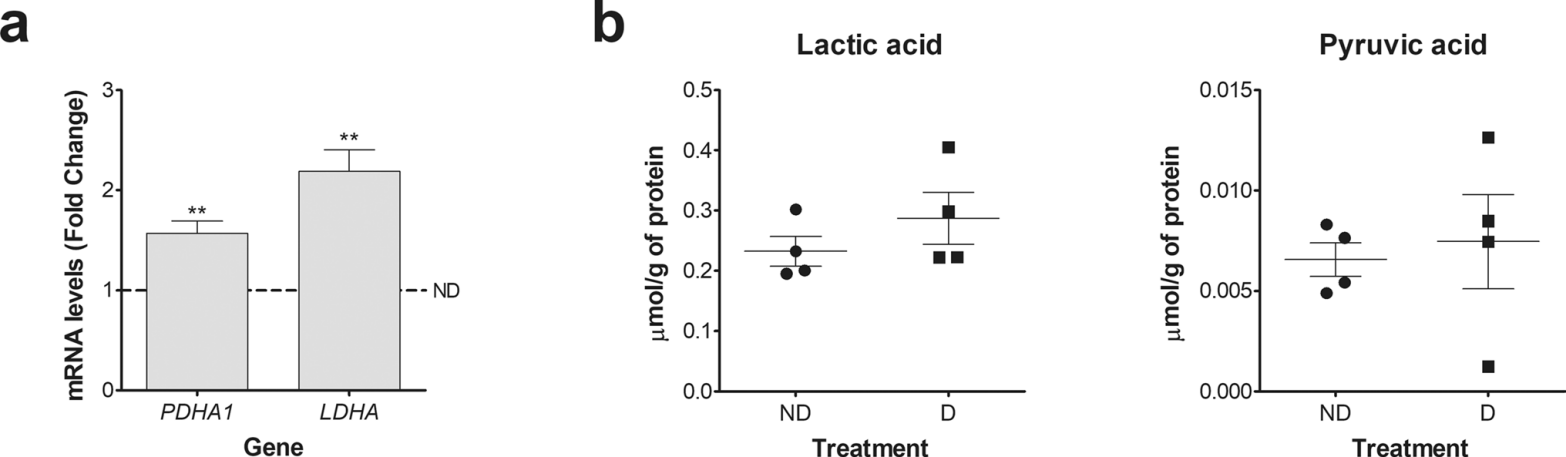
In vitro decidualization of HESC modifies the expression levels of enzymes associated with pyruvic and lactic acid metabolism. HESC were cultivated for 6 days in 2%-FBS medium containing MPA, E, and 8-Br-cAMP (D cells) or in 2%FBS medium containing vehicle solutions (ND cells). **a** *PDHA1* and *LDHA* mRNA expression levels. Data represent mean fold change compared to ND cells (dotted line, fold change = 1.0) ± SEM (*n* = 5). **b** Lactic and pyruvic acid levels. Data represent mean μmol/g of total protein (*n* = 4). **p < 0.01 (compared to ND cells). HESC, human endometrial stromal cells; D, decidualizing; ND, non-decidualizing; MPA, medroxyprogesterone acetate; E, estradiol; 8-Br-cAMP, 8-bromo-cyclic adenosine monophosphate; LDH, lactate dehydrogenase; and PDH, pyruvate dehydrogenase

The enzyme pyruvate dehydrogenase (PDH) converts the pyruvic acid produced during glycolysis into acetyl-coA, regulating the connection of glycolysis with the TCA cycle for efficient ATP production. Additionally, the pyruvic acid is reversibly converted into lactic acid by the action of the enzyme lactate dehydrogenase (LDH). Therefore, we evaluated the mRNA expression levels of *PDHA1* and *LDHA* and the content levels of lactic and pyruvic acid. The expression levels of the mRNA of both enzymes were upregulated in D compared to ND cells (Fig. 3a). However, even though we found differences in the expression levels of the enzymes involved in lactic and pyruvic acid formation, we found no differences in the content levels of these two metabolites between D and ND cells (Fig. 3b).

**Fig. 4:**
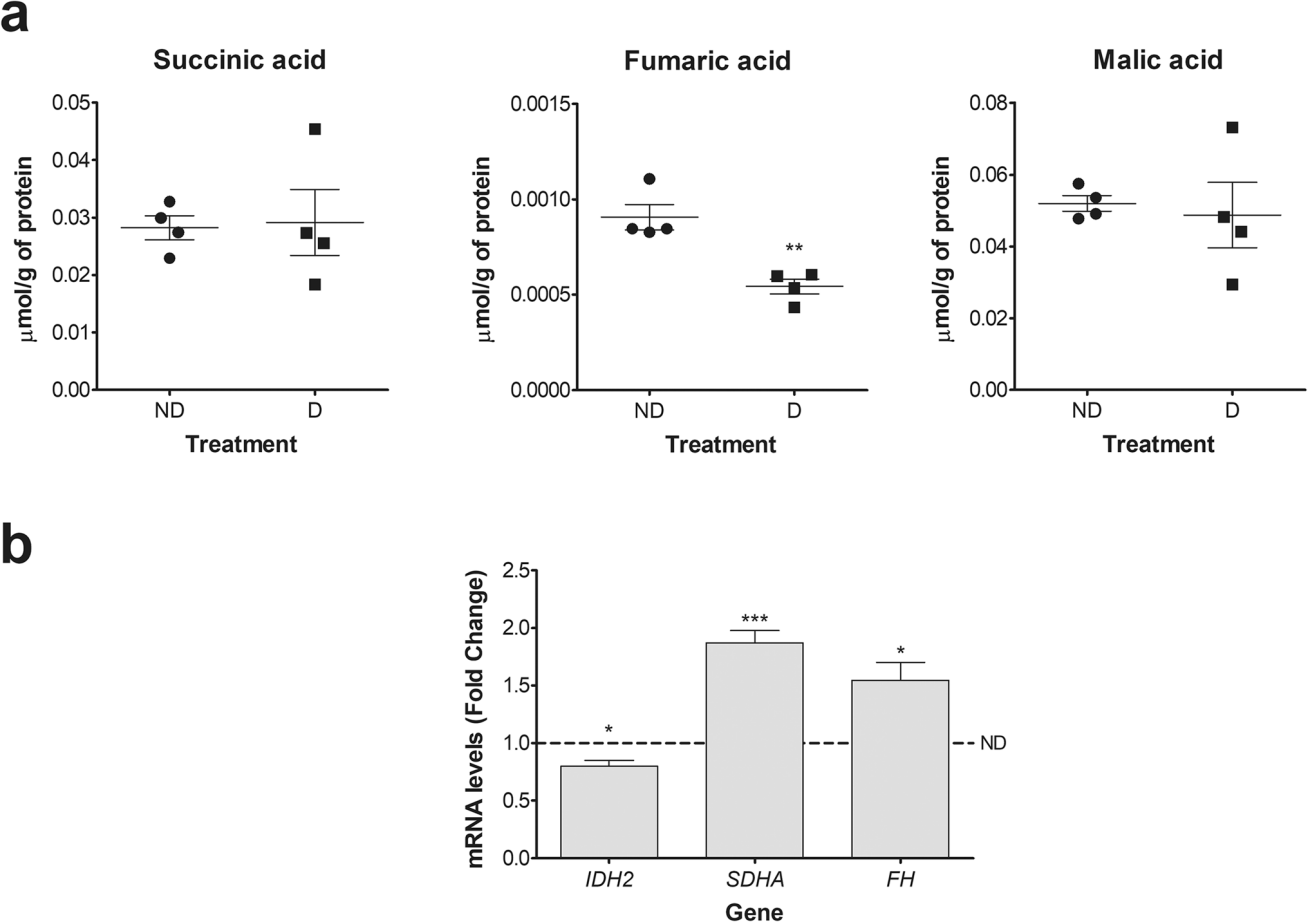
In vitro decidualization of HESC modifies the content of intermediate metabolites and the expression levels of enzymes associated with TCA cycle. HESC were cultivated for 6 days in 2%-FBS medium containing MPA, E, and 8-Br-cAMP (D cells) or in 2%FBS medium containing vehicle solutions (ND cells). **a** TCA cycle metabolites levels. Data represent mean μmol/g of total protein (*n* = 4). **b** *IDH2*, *SDHA****,*** and *FH* mRNA expression levels. Data represent mean fold change compared to ND cells (dotted line, fold change = 1.0) ± SEM (*n* = 5). *p < 0.05, **p < 0.01, and ***p < 0.001 (compared to ND cells). HESC, human endometrial stromal cells; D, decidualizing; ND, non-decidualizing; MPA, medroxyprogesterone acetate; E, estradiol; 8-Br-cAMP, 8-Bromo-cyclic adenosine monophosphate; IHD2, Isocitrate dehydrogenase; SDH, succinate dehydrogenase; and FH, Fumarate hydratase

Finally, as glycolysis and β-oxidation pathway final products converge in a common pathway, the TCA cycle, we evaluated the mRNA expression levels of three enzymes involved in the TCA cycle and the content levels of succinic, fumaric and malic acid, three intermediate metabolites of the TCA cycle. The enzymes analyses were isocitrate dehydrogenase 2 (*IDH2*), the enzyme that catalyzes the rate-limiting step of the cycle; succinate dehydrogenase A (*SDHA*), an enzyme part of the succinate dehydrogenase (complex II) that catalyzes the conversion of succinic acid into fumaric acid; and fumarate hydratase (*FH*), the enzyme that catalyzes the conversion of fumaric acid into malic acid (See Supplementary Fig. S1). Our analysis showed that the gene expression levels of *SDHA* and *FH* were upregulated in D cells compared to ND cells, in contrast, the gene expression levels of *IDH2* were slightly, still significantly, downregulated in D compared to ND cells (Fig. 4b). In regards to metabolites content, the level of fumaric acid was reduced in D compared to ND cells, while the level of succinic and malic acid had no differences between D and ND cells (Fig. 4a).

## Discussion

In this study, we show that decidualizing and non-decidualizing HESC have differences in the mRNA expression levels of key enzymes and in the content level of the different metabolites of glucose and fatty acids catabolic pathways: glycolysis, PPP, β-oxidation pathway and TCA cycle.

### Glucose transporters, glucose catabolism and in vitro decidualization of HESC

First, in this work, we validated the upregulation of *GLUT1* mRNA expression during in vitro decidualization of HESC. In addition, we found no difference in *GLUT3* mRNA expression. These results are in line with previous studies which showed that meanwhile GLUT1 increased expression was dependent on endometrial stromal cells, GLUT3 increased expression was dependent on CD45+ immune cells in the human decidua [9]. Overall, these results strongly suggest that GLUT1 is the main glucose transporter used during decidualization by ESC. In relation to glucose catabolism, we found an upregulation of *G6PD* mRNA expression, reinforcing the importance of the PPP pathway during in vitro decidualization of primary HESC in accordance with results previously described by Frolova et al. [12]. To note, one of the main products of the PPP pathway is the ribonucleosides (RN) and deoxyribonucleosides (DRN) precursor, ribose-5-phosphate. It has been shown that the addition of RN and DRN recovers the reduced in vitro decidualization of immortalized HESC (t-HESC) produced by PPP inhibitors (DHEA and 6AN) [12]. This effect was dependent on RN and DRN dose and on the PPP inhibitor used. Low and high doses of RN and DRN reverted PRL and IGFBP1 expression in cells treated with 6AN. However, only high doses of RN and DRN reverted PRL but not IGFBP1 expression in cells treated with DHEA. Those results by Frolova et al. highlight that the interconnection of glucose catabolic pathway with other important cellular metabolic pathways is essential for the correct decidualization of ESC [12].

In terms of glycolysis, there are controversies in regard to its importance for the proper decidualization of ESC [11–13, 20]. Taken together, in our work, the upregulation of *PDHA1* mRNA levels and the constant levels of pyruvic acid content between D and ND cells could suggest that glycolysis is required and more active during decidualization, as previously reported [11, 20], and that the pyruvic acid produced during glycolysis is being rapidly redirected to acetyl-CoA production in D cells. Additionally, the upregulation of *LDHA* mRNA levels could suggest that the pyruvic acid, product of active glycolysis, could be similarly rapidly transformed into lactic acid, used by dividing ESC, as previously described [11]. This connection between dividing and decidualizing cells through lactic acid utilization could explain why we found no differences in lactic acid content levels between D and ND cells. Our results, highlight the importance of the interconnection of different metabolic pathways within the cell, comprising that the recently produced products are constantly incorporated into related metabolic pathways.

### Fatty acids catabolism and in vitro decidualization of HESC

As we show in this study, the mRNA expression levels of *CPTA1* and *CPT2* were upregulated in D compared to ND cells, suggesting a more active β-oxidation pathway during in vitro decidualization of HESC. These results agree with previous data that highlight the importance of the β-oxidation pathway for the correct decidualization of ESC [17]. In addition, it has been described that in breast cancer cells prolactin increases the expression and the activity of CPTA1 in relation to normal cells [21]. The mammary gland and the endometrium are both hormone-responsive tissue, that share also the expression of PRL receptors. In our study, ESC increased PRL and *CPTA1* expression in response to the decidualization stimuli. Further studies are needed to define if *CPTA1* mRNA increased expression is linked to PRL increase or if another signaling pathway is responsible for their increased expression in ESC during decidualization. Moving forward to the evaluation of the intermediate metabolites of the β-oxidation pathway in our study, unexpectedly, the content levels of C2- and C4-acylcarnitines were decreased in D compared to ND cells. Interestingly, among all the different β-oxidation metabolites, C2-acylcarnitine is closely related to the content levels of acetyl-CoA produced in the mitochondria, as acetyl-CoA needs to be bound to carnitine for its transport outside the mitochondria as C2-acylcarntitine [14–16]. Therefore, a low presence of C2-acylcarnitine in decidualizing ESC represents a low intracellular content of mitochondrial acetyl-CoA as well.

Even though the results regarding *CPTA1* and *CPT*2 mRNA expression levels and C2-acylcarnitine content seem contradictory, the interconnection of metabolic pathways could explain a fast uptake of the acetyl-CoA produced by a more active β-oxidation of fatty acids during decidualization. It is known that acetyl-CoA is a key metabolite that interconnects different metabolic pathways of great interest for cellular metabolism and function [22]. In this sense, acetyl-CoA also has an important role as a precursor of anabolic reactions, like lipid, steroid, and specific amino acid synthesis. Furthermore, acetyl CoA contributes to protein acetylation, including histone acetylation in the nucleus and protein acetylation in the cytoplasm and mitochondria, and it also serves as an allosteric regulator of the activity of several enzymes. It has been described that a low glucose environment affects the acetylation levels (reduced H3K27ac levels) of the promoter regions of certain genes involved in decidualization, like PRL, IGFBP1, and FOXO1 [23]. Those authors have suggested that the lower acetylation could depend on the lower levels of acetyl-CoA resulted from the low-glucose environment.

### TCA cycle and in vitro decidualization of HESC

As a main metabolic pathway related to energy production, the incorporation of acetyl-CoA, produced from fatty acids and also from glucose, into the TCA cycle and the mitochondrial electron transport chain is of great importance for cellular function. Therefore, moving forward in the interconnected catabolic routes, by the results obtained in this work, it is difficult to define whether the TCA cycle is more active in D compared to ND cells. By the expression levels of the enzymes, we could interpret that the overall TCA pathway seems to be less active during decidualization (Fig. 4). The result that supports this is the reduced expression levels of *IHD2*, the rate-limiting enzyme of the TCA cycle, which is slightly down-regulated in D compared to ND cells. Conversely, *SDHA* and *FH* mRNA levels were upregulated in D compared to ND cells, suggesting that the last part of the cycle could be more active than the first one. These results could implicate that the intermediate metabolites from other metabolic pathways, independent from glucose and fatty acids, could be joining the TCA cycle. Therefore, in relation to the intermediate metabolites, we found a different pattern compared to the corresponding expression levels of the enzymes, as the intermediate metabolites malic and succinic acid had no changes between D and ND cells. This could also be interpreted as an overall steady-state content of intermediate metabolites by a derivation of the intermediate metabolites to other metabolic routes. Further studies are needed to define the difference in the activation of TCA cycle in D compared to ND cells.

Rhee et al*.* reported that the ATP concentration is lower in D than in ND-immortalized human ESC (t-HESC) [24]. It is, therefore, in agreement with our findings that the connection of the TCA cycle with the mitochondrial electron transport chain to produce high amounts of ATP, could be redirected to interconnect the intermediate metabolites of glucose and fatty acid catabolism with a less active TCA cycle and other metabolic pathways during decidualization. In addition, Rhee et al*.* suggest in their publication that low levels of ATP in decidualizing cells may activate AMPK, an AMP/ATP sensor kinase known to activate autophagy, as two targets of AMPK are phosphorylated during decidualization of t-HESC [24]. We have previously shown that autophagy is increased and that it is important for the proper decidualization of t-HESC, as the knock-down (KD) of two autophagy-related proteins, ATG5 and ATG7, impairs the in vitro decidualization of t-HESC [8]. The importance of autophagy for decidualization was additionally validated by other research groups using in vitro and in vivo mice models of decidualization [25, 26].

## Conclusions

The energetic metabolism of cells depends on various pathways that are all interconnected through their intermediate metabolites and final products, for the proper regulation of the catabolic and anabolic processes needed for optimal cellular function and survival. Furthermore, most of the metabolic enzymes of the main catabolic and anabolic pathways of the cell are bi-directional, adding another challenge to the evaluation of its activation by the expression levels of its main enzymes.

Taken together, the findings presented here suggest that HESC undergo energetic metabolic changes during decidualization and that D and ND cells differ in the level of activation of different metabolic pathways and in the use of intermediate metabolites. Further studies are needed to define with precision how the different metabolic pathways are interconnected and which ones are most important during the decidualization process of ESC and which metabolic differences affect the functionality of decidualizing and non-decidualizing ESC.

The continuous study of the interconnection of metabolic pathways in the endometrium will allow the development of new therapeutic approaches based on the administration of key intermediate metabolites to contribute to the proper decidualization during the implementation of assisted reproductive technologies. We are positive that the better understanding of the energetic metabolism during decidualization will contribute to improve women’s reproductive health.

## Supplementary Information

Supplemental Table S1 (PDF 31 kb)

Supplemental Figure S1 (PNG 592 kb)

High Resolution Image (TIF 1876 kb)

## Data Availability

Data will be provided upon the request.

## Abbreviations

*6AN*: 6-aminonicotinamide
*ATG*: Autophagy-related
*8-Br-cAMP*: 8-Bromo-cyclic adenosine monophosphate
*C2*: Acetyl-acylcarnitine
*C4*: Butyryl-acylcarnitine
*CoA*: Coenzyme A
*CPT*: Carnitine Palmitoyl Transferase
*D*: Decidualizing
*DHEA*: Dehydroepiandrosterone
*DRN*: Deoxyribonucleosides
*E*: Estradiol
*ESC*: Endometrial stromal cells
*ESI-MS/MS*: Electrospray Ionization-Tandem Mass Spectrometry
*FH*: Fumarate hydratase
*G6PD*: Glucose-6-phosphate dehydrogenase
*GC-MS*: Gas Chromatography-Mass Spectroscopy
*GLUT*: Glucose transporter
*HESC*: Human ESC
*IDH*: Isocitrate dehydrogenase
*IGFBP1*: Insulin-like growth factor binding protein 1
*LDH*: Lactate dehydrogenase
*MPA*: Medroxyprogesterone acetate
*ND*: Non-decidualizing
*PDH*: Pyruvate dehydrogenase
*PPP*: Pentose phosphate pathway
*PRL*: Prolactin
*RN*: Ribonucleosides
*SDH*: Succinate dehydrogenase
*SEM*: Standard error of the mean
*TCA*: Tricarboxylic acid
