# Supplementary material for: Glucose and fatty acids catabolism during in vitro decidualization of human endometrial stromal cells
Source: J Assist Reprod Genet. 2022 Oct 29;39(12):2689–97. doi: 10.1007/s10815-022-02637-3 (PMC9790837; doi:10.1007/s10815-022-02637-3)
Supplement: Supplementary file 1 — (PDF 31 kb) [file 10815_2022_2637_MOESM1_ESM.pdf]

Gene	Catalog Number
IHD2	Hs00953879_m1
SDHA	Hs00188166_m1
FH	Hs00264683_m1
PDHA1	Hs01049345_g1
LDHA	Hs01378790_g1
CTP1A	Hs00912671_m1
CPT2	Hs00988962_m1
GLUT1	Hs00892681_m1
GLUT3	Hs00359840_m1
G6PD	Hs00166169_m1

Supplemental Table S1. Detailed list of primers used (Taqman technology, Life Technologies GmbH, Darmstadt, Germany).
