# Supplementary figures and images for: Glucose and fatty acids catabolism during in vitro decidualization of human endometrial stromal cells
Source: J Assist Reprod Genet. 2022 Oct 29;39(12):2689–97. doi: 10.1007/s10815-022-02637-3 (PMC9790837; doi:10.1007/s10815-022-02637-3)

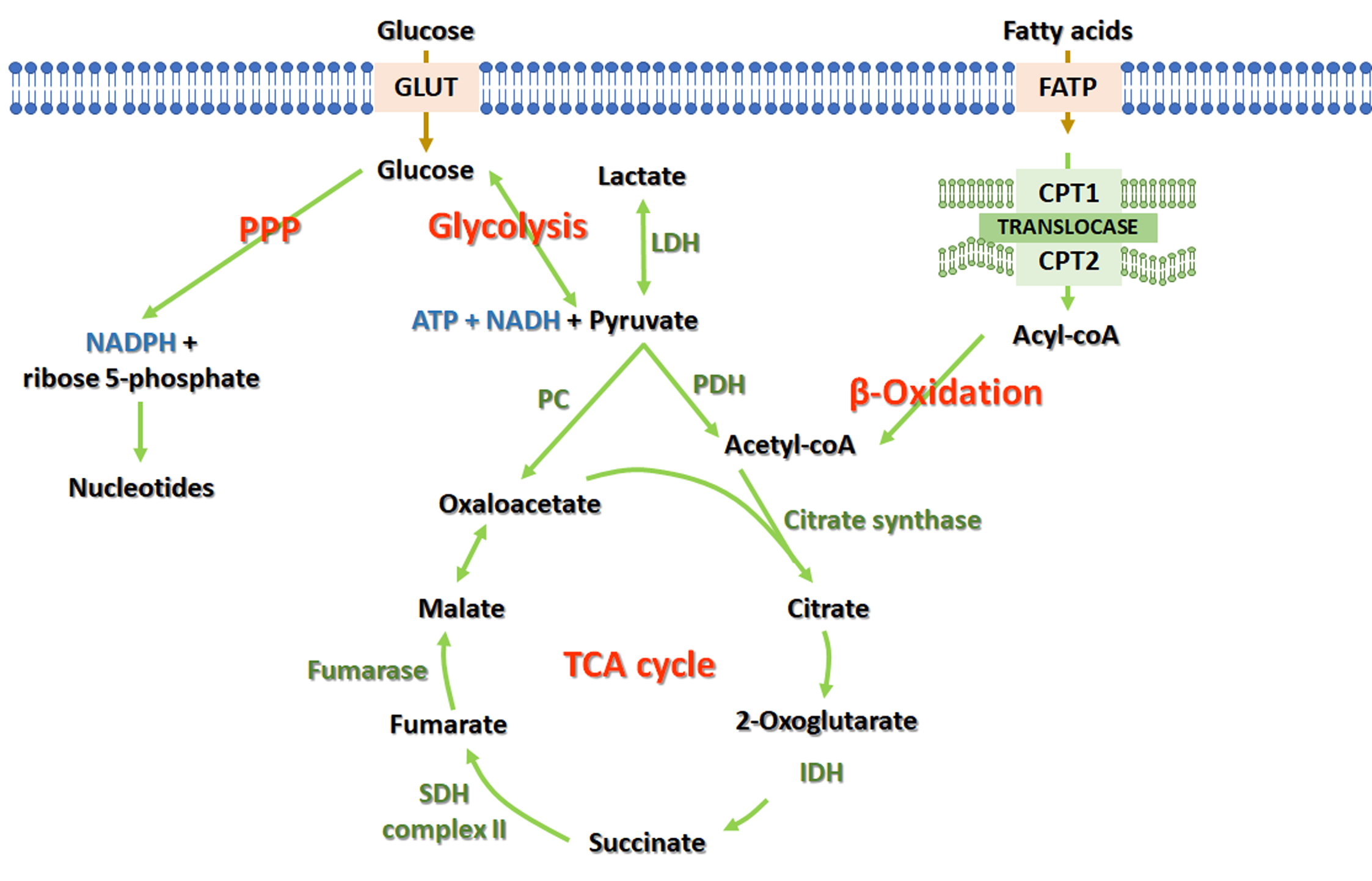

Supplement: Supplementary file 2 — (PNG 592 kb) [file 10815_2022_2637_Fig5_ESM.png]
